# How Can We Magnify the Importance of Our Calling?

**Published:** 1892-11

**Authors:** G. A. Mills

**Affiliations:** New York City


### HOW CAN WE MAGNIFY THE IMPORTANCE OF OUR CALLING?[1]

BY G. A. MILLS, NEW YORK CITY.

Coming as I did from a Massachusetts inland town to New York City, May, 1861, and imbued with a sincere longing for a something allied to professional attainments that I did not possess, I was by virtue of such circumstances ready to accept any proffered association that might lead to the satisfaction of my desire.

During my entire adult life I have found that it is the most unexpected that comes to us, and in no part of my career has it been more noticeable than in my professional life.

Dr. W. H. Atkinson, by a very providential experience, was also directed to New York City in June, 1861. That his coming has proved a valuable event to the entire dental profession hardly needs any emphasis from me.

This was seemingly a novel transit for such a character, so unconventional, to be transplanted into a metropolitan atmosphere.

---

[1] Read before the California State Dental Association, July 22, 1892.

Very few of the dental profession in New York City, then including the prominent names of Drs. Parmley, Dwinelle, Duning, Rich, Foster, and Dodge, gave him any recognition; these practitioners stood for all that was thought to be the standard. Dr. Atkinson's coveted endowment was a fraternal nature, and with it the unusual accompaniment of mental capacity that has known no equal in our ranks. He was truly a "teacher of teachers." These characteristics soon surrounded him with a class of listeners. In those days there were some that called him egotistical, but those now living can recall how childlike he seemed as they gathered eagerly about him and assimilated his instructions.

Dr. Atkinson's advent and its success was certainly phenomenal, both in his finding earnest listeners and a full demand for his services. This last encouragement was largely added to by those that came to witness his clinics, which were given on each Monday forenoon. At these clinics he never failed for need of patients, for he could always find a suitable case among his audience which he could use as valuable instruction. The novelty first introduced by him was the hand-mallet, which was truly an innovation, but adopted almost by acclamation. It was then an ivory one: note the many devices since. Cohesive gold added another attraction; although its use was not unknown, yet it had not been generally adopted. Contouring teeth deformed by caries was still another. He carried war into the common practice of deforming teeth by file and chisel, and denounced this as a sacrilege. While the method of contouring teeth had become a part of our literature, yet it had at this period taken but little hold of general practice. True in all his observations in nature, he called attention to the fact that to contour a tooth did not supply the demand of nature's use in a proper mastication. He demonstrated her needs in the mouth,—viz., that lateral support throughout the entire arch was required. From this method originated the term "knuckling" teeth together. This idea was not easy to inculcate in the minds of many, for the prevailing practice of "self-cleaning spaces" predominated. It was for this purpose that he introduced the wood matrix to exaggerate the space, that he might secure the firm support of tooth to tooth. Who will say at this day that he wrought not wisely and well in suggesting this valuable method, so largely recognized now by good operators, and for the lack of which much discomfort and loss of valuable teeth has resulted?

I need only recur to these things to make the record true, and pass on without further detail, except to notice, for the benefit of young practitioners, the fact that this radical reform was commenced without the use of dental engines and rubber dam. Think of the amount of hard, close, confining labor performed by hand instruments. This was in the spring of 1862, and rubber dam came in 1866, the engine in 1870.

We have gone over glibly this first step in an out-and-out reform, which has come to remain as long as teeth need repair by the facilities now known. At this time disordered conditions of teeth were not looked upon as in any large degree amenable to surgical dealing with faith in their future usefulness. "You can deal successfully with alveolar abscesses, acute and chronic, with as much hope of success as with any other part of the body," was emphasized so confidently by Dr. Atkinson that many a good practitioner replied, with a quick breath, "That is just what we all want to know how to do;" and just here he saw the necessity for foundation-teachings. Many of us will recall his familiar manner of accepting all men at first call, as knowing something of the things he was dealing with, yet often, finding how little was known, he would turn the major part of his interview into a teaching class. From the start he found it absolutely impossible to progress with his clinical cases without prefacing them with instructions. Sometimes his dealings were gentle, and then the opposite, for he would not let any one pass himself off for what he was not without trying, at least, to put him on the track of intelligence, regarding it as true friendship to lead one away from an error. What an example worthy of our best emulation!

Returning again to the surgical treatment of alveolar abscess. Heroic attention from the start: creosote, No. 1, iodine, No. 2,—a saturated solution of crystallized iodine and creosote, half-and-half. Tannin and glycerin, No. 3; put all the tannin into a portion of glycerin that it would take up, making a stiff ointment. What have we got in the place of these? If dead bone were associated, officinal preparations of aromatic sulphuric acid were used; if superficial, the burr was called upon to remove it. This we will refer to later as the introduction of a systematic method dealing with surgical territory.

We return again to the application of the wood matrix. The idea was to get direct access to all cavities, so far as practicable, and, by the aid of wedge or matrix, to arrive at a proximal cavity direct through the grinding surfaces. This was in direct opposition to the then common mode of procedure. To reach these obscure decays space was made by rubber pressure, which he condemned wholly, claiming that quick and rapid moving of teeth was the safest and most humane. It is easy to review, by a little activity of the mind, how evolution has carried us on. But all these facts will show that he was working a decided reform in dental practice, which was constantly italicizing the importance of saving teeth in a far larger sense than any practitioner had ever before advocated, and out of his intensity originated the saying that followed him ever after, "To extract a tooth is an evidence of disability to save it." Is it not true to-day?

I cannot attempt to lay before your thought the multiplied detail of reminiscences that crowd upon me, so I must part company and press on to those things that are to be made to emphasize the subject I have in mind. It soon came to pass that Dr. Atkinson's field was too circumscribed for his growing enthusiasm, and with his nucleus of ardent followers, he at the head and front always, came the formation of associations. The first body of this kind that had captured the attention of progressive men was the well-known society, the Brooklyn Dental Association. It was in this society that we first met the delegation from California. If I recall rightly, the late Dr. Dutch was the first on the roll, for every visitor with any purpose was made a member, Dr. Knowles being the next in order. This must not be confounded with the Brooklyn Dental Society, for that body was not in existence before 1866.

The Brooklyn Dental Association held a name to live, and it did live, and it made alive all members that came in touch with it. What characterized it so much above anything before was its fraternal nature, generated by Dr. Atkinson alone. He became the moving and guiding factor with the entire body. For membership it knew no particular locality, only that the person was in pursuit of knowledge. With Dr. Atkinson a dentist was a dentist, and he did not feel that he bemeaned himself that he aided the unwashed. Some men that he has helped and made fit for better things may have forgotten the pit from which he did more than any other to dig them and to put them into a position of usefulness. Do they show themselves as liberal towards their needy brothers?

With the growth of liberal associations Dr. Atkinson's opportunities widened for the greater work for which he was fitly prepared; we add, chosen, and no meeting was complete until his voice had been heard. How many are the occasions that we can recall that he awakened us to a high pitch by his enthusiastic fervor! Many said he talked so high into the air that common mortals could never hope to follow him. How is it now? Are we not following him in many ways more than we think, or even know, to-day? A consensus of his writings would show us how definitely he has outlined the path of scientific attainment for future advancement. They are replete with the discussions of basal principles, without a knowledge of which we cannot hope to build an enduring work. His articles upon Histology, Microscopy, Inflammation, the Origin of Pus, are rich with information, and he who becomes most able to assimilate such will become more a master of his calling. These articles are truly the whisperings of "the angels." Note this, they will never need any revision from us. A pet idea of his, he did not live to see put into practical operation,—viz., teaching the student over the case; telling to each one, directly, his own needs. Professor Truman has hinted his leaning in this direction. The school that first puts it into operation will set a potent example for good, and a lasting benefit to our calling. It will give education such an impetus as it has never known. Preaching dentistry by platform oratory is too stale for any one who has a mission in life to be patient enough to wait to hear. Whatever some may think of what have been termed vagaries of Dr. Atkinson, his spirit has become infectious, and the work of the future will be more zealous because of it.

I have only lightly surveyed this hurried work of his, as he has come and gone before us, so often in his quickened movements, yet even this will serve to inspire us in the retrospect. One point I desire to emphasize, so that it may become indelible in all minds; viz., thirty years have passed, and Dr. Atkinson's name stands, and must ever stand, as the founder of an advanced school of practice in dentistry.

I have gone over principally the mechanical phase, as this was more in the minds of his first admirers, and by this the door was effectually opened to emphasize the larger thoughts which were only waiting to be brought forth. The advancement of the views of such greater possibilities in the saving of teeth produced a suitable soil for the advocacy of a practice beyond anything before thought of, and this doctrine became imbued in the minds of wise-thinking practitioners. Dr. A. C. Hawes was inspired to offer that effectual resolution in the Brooklyn Dental Association, calling the serious attention to the ruthless destruction of the natural teeth by extraction. Compare the value of a human tooth then and now. In the light of the minute anatomy as given us by Heitzmann and Bödecker, we find it an object worthy of worship.

In this connection, Dr. Atkinson's active interests were almost unbounded, for he found in the "grand man," as he termed him (Heitzmann), a co-worker whom he had hardly hoped to discover. In him he recognized the "missing link" that he had so longed for in his chain of personal observation and investigation, and no one better knows than myself the ardor that he applied in forming the first classes for microscopical study of the "morphology of tissues" in Heitzmann's laboratory.

No child was ever more enthusiastic over a new toy than Dr. Atkinson in that first class. We can hear him now exclaiming as each beautiful new definition was shown under the lens and so intelligently explained by the professor. He, too, caught the childish glee and carried no little of it into the spirit of his teachings; and why not? He had never known such cordial acknowledgment, and it became such an inspiration as he had never before met. With this work has dawned upon our calling a true scientific sky, and, true to the last, Dr. Atkinson, in the last speech he made before the Odontological Society eulogized Heitzmann's reticulum theory. It was a tribute from a dying hero to a living hero in science. Dr. Atkinson has often said this work of Heitzmann's settles the question of a sure scientific foundation for our calling. This has crystallized into a concrete practice, which he had seen in his inspirations; and now they are ours in pages of books to remain. All honor to him who was permitted to see his desires realized. Who can say that Dr. Atkinson's life was a failure? Weak men have emphasized it, and, more, there are those right here in New York that disgrace themselves by defaming his memory. Shame! shame! that men can so degrade themselves.

In no one field have the teachings of Dr. Atkinson been more pre-eminently practical than in surgery. By his example he has reduced much of what had formerly become major surgery to minor surgery. His views were antagonistic to what was termed the "expectant treatment." For instance, if necrosis was manifested, nothing was thought of being done to conserve the parts, "but it must be left for the system to deal with it, trusting that a sequestrum would be the result." What may be called minor dealings in a wide range of lesions have been established as surgery in the chair, and robbed of its terrors. It is no longer an assumption to say that in a dental chair operations are daily performed that are of incalculable service to humanity, and thus we are elevated in the scientific estimation, and the importance of our profession is magnified.

In the next quarter of the century the rightly-educated practitioner will be in demand to a degree as never before. This new departure in teaching has enhanced the possibilities of our usefulness that should create a fervor that will do honor to the memory of him who has been inspired to make it possible.

If I refer to the remunerative advancement this advanced field of labor has produced, it will be more seen in a practical sense. Surgery by one that is properly educated commands an attention and a fee that thirty years ago were not known or thought of. This class of service is doing away with "jobs," and an intelligent public are finding out that sickness of tissues is not to be corrected in that way. And we may ultimately hope to see the time when it will no longer be rung in our ears, "What do you charge?" but the first thought will be, Is he skilful?

At no time in our history has there been more serious thought for the future good of our calling, and we all agree that we think it timely that it is so. The *Ohio Dental Journal* has recently asked a question that should have our wisest consideration. Why do so few young practitioners attend society meetings? This question should not only be asked by all thoughtful practitioners, but it should become the business of all to give it earnest study and find out why, and set about seeing that it is corrected. It will be of value to study the age in which we are living. Dr. Morgan Howe said not long since, at one of the meetings of the Odontological Society, that it seemed to him that too many of our young practitioners were manifesting too large an interest in the financial part of practice. There will be those that will say that in this they think they are wise. Financial interests are not to be ignored by any means, but an over-attention in this direction will work ill to the best interests of our calling.

Close readers of the journals have noticed that some have discussed finances, and have originated the idea that more money is to be made in some other calling. True, this may be. How comes this? Those that are saying these things are reputed as moneyed men; some of the same men are congratulating themselves that their sons have not joined the ranks of dentists. We do not wish to try and analyze the meaning of such statements, even if we could.

Every year makes it more apparent that our calling is of no mean character, and one that is to call for a standard of ability which will demand investigations, aided by energetic and scientific co-laborers, equal to any other calling, and I do not think I overestimate the true situation when I say that to meet the demand it will require men fitted with an education of hand and head, and I may rightly add heart, without which no calling of need is worthily followed to the uttermost. Professor Miller has not emphasized the importance of our work too highly in the beginning of his late article in the *Dental Cosmos.* The mouth, our field, is far from being thoroughly understood. We are but upon the threshold of a scientific survey of its real needs. The only real scientific knowledge we have of it is the minute anatomy of the teeth. Of the fluids that are associated with their internal and external conditions we know little or nothing, and possess but an empirical sense of their action and reaction, of the changes that are occurring from time to time, and that these are liable to take place by sudden environments that come and bring serious results to the structural character of the teeth and their allied tissues.

What is our knowledge of the waste of the external portions of teeth which we term erosion and abrasion? Are they one and the same? What is our knowledge of the most serious and destructive conditions that we are witnessing daily, so-called "Riggs's disease" and "pyorrhœa"? Who are the ones to give us the true etiology of these disorders?

Let those who treat lightly scientific investigations answer these questions, if they have the brain. In the light of these queries, let him that has a particular gift apply himself to it with an arduous devotion, and each respect the zeal of the other and vie with each for a place in the front ranks. Such opportunities as are offered to young practitioners in our calling, in our estimation, are rare. To aim high means a purpose to attain to things above the ordinary. It is in our thought to be able to stimulate mental action in the direction of magnifying the importance of our work. We have large faith in the future of our advancement to a position that will ultimately place us beside the more exalted. For this, the late Dr. Atkinson zealously labored and spent himself to the end of his career. In the last few months his very earnest thought dwelt upon the subject of adenoid growths. It was his daily study to add a new knowledge to the subject so little understood from an etiological view. Had he remained with us until the May meeting of the Amerian Medical Association he would have presented us a valuable consensus of his thoughts upon the subject.

Many times has it been remarked as strange that he had never given us a text-book of any kind, and often has he been heard to say "he was glad that he had never fallen into such a folly."

Dr. Atkinson's work was to inspire men to thought formed upon basal principles. He often grieved at the lack among his fellows of mental philosophy. But he did not go from among us without a lively feeling of encouragement that he saw a better prospect for the coming days. It was often repeated in our hearing, "the boys are growing; they are getting a grasp of things that will not permit of the domination of ignorance as in the past." The new school of practice calls for this class of thinking. Our thinking must be on the basis of mental philosophy to have it intellectual. It is coming to be a lamentable fact, and it is the cause of much intelligent discussion that is recorded in our monthly literature. If our discussions were so formed as to show how men arrived at their conclusions, this great error, so common, would soon be corrected; until it is, it will be a serious hinderance to our advancement.

Men cannot deal with surgery on the basis they do with mechanics. It demands a grade of mental action that must be intelligently associated with intuition,—possibly psychology may better express my meaning. It may often occur that a good mechanic may be destitute of this higher quality of ability. That it may be cultivated, I think, is true, yet it is in larger ability an inheritance or gift. So it may be that one will be largely qualified in the latter quality and largely deficient in the former. At this point of thinking some will inquire, How can we ever hope to make a practical division of our calling? All along these lines of thought are open fields for emphasizing its importance.

We can, under the inspiration of this subject, put our minds into active research for examples that tell us that there has been a quality of intellect working in this direction, and we are to see more of it,—such bright intellects as the author who wrote that unanswerable article for the *Dental Cosmos* on the ethics of law (and the late Dr. James White honored it with an editorial), Thompson, of Topeka, Kansas; Talbot, of Chicago; Ingersoll, Kingsley, Andrews, Bödecker, J. L. Williams, now of London, Barrett, and others. The labor of these men is forming a composite without which no lasting foundation could be formed, and in the same line all of our brilliant practical workers are nobly filling a niche of no mean importance. Also, every earnest, sincere laborer is a valuable contribution, however humble he may think himself to be. Work for all, only work while it is day; here a little and there a little, carrying each day the ambition a degree higher than yesterday. In this way, more and more will our work be emphasized. And, lastly, our prime thought must be to be of use in its highest sense and to those we are called to serve. In this way we will answer the question, and in the most emphatic and practical manner, How can we educate the people? The response will be, Take our highest standard for those in need as they come to each one of us day by day,—not those of our neighbor, but those that divinely seek us for that which we have been sent to do. Whose professional character can we more profitably photograph upon our daily mission than that of the noble, heroic, unselfish, fraternal Atkinson? I cannot conceive of a pattern that can prove of so much stimulation to the true professional gentleman. Having been faithful to the light we have had bestowed upon us, we will be able to say at the close of an active, useful, humane mission, *Exegi monumentum ære perennius.*

